# Nicotinamide Efficiently Suppresses Porcine Epidemic Diarrhea Virus and Porcine Deltacoronavirus Replication

**DOI:** 10.3390/v15071591

**Published:** 2023-07-21

**Authors:** Mingxia Li, Liping Zhang, Li Pan, Peng Zhou, Ruiming Yu, Zhongwang Zhang, Jianliang Lv, Huichen Guo, Yonglu Wang, Sa Xiao, Xinsheng Liu

**Affiliations:** 1 State Key Laboratory for Animal Disease Control and Prevention, Key Laboratory of Animal Virology of Ministry of Agriculture, Lanzhou Veterinary Research Institute, Chinese Academy of Agricultural Sciences, Lanzhou 730046, China; 15730972905@126.com (M.L.);; 2 College of Veterinary Medicine, Northwest A&F University, Yangling 712100, China

**Keywords:** nicotinamide, porcine epidemic diarrhea virus, porcine deltacoronavirus, replication

## Abstract

Porcine epidemic diarrhea virus (PEDV) and porcine deltacoronavirus (PDCoV), members of the genus Coronavirus, mainly cause acute diarrhea, vomiting and dehydration in piglets, and thus lead to serious economic losses. In this study, we investigated the effects of nicotinamide (NAM) on PEDV and PDCoV replication and found that NAM treatment significantly inhibited PEDV and PDCoV reproduction. Moreover, NAM plays an important role in replication processes. NAM primarily inhibited PEDV and PDCoV RNA and protein synthesis rather than other processes. Furthermore, we discovered that NAM treatment likely inhibits the replication of PEDV and PDCoV by downregulating the expression of transcription factors through activation of the ERK1/2/MAPK pathway. Overall, this study is the first to suggest that NAM might be not only an important antiviral factor for swine intestinal coronavirus, but also a potential candidate to be evaluated in the context of other human and animal coronaviruses.

## 1. Introduction

PEDV, an enveloped and single-stranded RNA virus, causes vomiting, diarrhea and a high mortality rate in neonatal pigs. PED was first reported in Belgium in the late 1970s and in the United Kingdom, and then spread globally, causing large economic losses [1,2]. PDCoV is a member of the genus Deltacoronavirus and was first reported in Hong Kong, China, in 2012 [3]. PDCoV is a novel swine enteropathogenic coronavirus that causes acute diarrhea, vomiting, and dehydration in neonatal piglets [4]. The clinical symptoms of PDCoV infection are milder than those of PEDV infection, and the mortality rate of PDCoV infection is approximately 40–50% [5]. To date, there are no commercial vaccines available for PDCoV [6,7]. In addition, virus neutralization cross-reactivity between PEDV and PDCoV has not been detected.

Nicotinamide (NAM), an amide form of vitamin B3, has been shown to be a nutrient in mammals [8] and is a component of coenzymes I and II. NAM is also a precursor for the coenzyme NAD+, which is necessary for the synthesis of nicotinamide adenine dinucleotide phosphate (NADP+) [9,10]. Through the tricarboxylic acid cycle, NAM participates in energy metabolism and utilizes NAD+ in the mitochondrial respiratory electron transport chain for the production of ATP [11]. This nutrient may prevent skin cancer because it can act against changes due to skin aging and tumor development [12,13]. Sirtuin 1 (SIRT1) participates in modifying the activities of various target proteins through deacetylation, and mediates key processes such as cell division, differentiation and aging to help cells survive under various stress conditions; and NAM is the most widely used SIRT1 inhibitor [14]. In addition, NAM is intimately related to pathways involved in apoptosis, autophagy and oxidative stress [15]. As a feedback inhibitor of poly (ADP-ribose) polymerase (PARPs), NAM acts as an antioxidant and plays a therapeutic role in inflammation-related diseases [16,17]. NAM also protects cells with severe DNA damage from PARP-induced apoptosis or necrosis caused by the depletion of NAD+ and ATP reserves [16]. Moreover, studies have indicated that NAM is involved in the maintenance of genome stability. In animal models, NAD+ deficiency impairs the DNA damage response, destabilizes genome stability and increases the incidence of cancer [13].

Many previous studies have shown that NAM plays an important role in protecting against infectious diseases [18,19,20]. NAM is a bioactive molecule with activity against *M. tuberculosis* and HIV [18,19]. Moreover, NAM can effectively inhibit HBV activity by reducing the activity of the HBV promoter and suppressing the replication of the viral genome [20]. NAM also prevents vaccinia virus core precursor peptides (P94 and P65) from being cleaved into mature core proteins to reduce the number of infections [21]. During adenovirus replication, NAM can be used as a competitive inhibitor of adenosine diphosphate (ADP) ribosylation to inhibit viral replication [22]. In addition, NAD+ and its intermediate (NMN) were found to protect 30% of aged mice infected with lethal mouse-adapted SARS-CoV-2 from death, so clinical trials will be conducted on the basis of these experimental results in mice [23]. In short, NAM is an effective antiviral agent, but its additional effects on other viruses still need to be further explored.

Mitogen-activated protein kinase (MAPK) acts as a global regulator of extracellular stimuli, and is an important signaling pathway associated with viral infection. This pathway may regulate viral infection through one or multiple steps in the infection cycle [24]. To optimize their replication, some viruses exploit this pathway [25]. Many drugs exert antiviral effects by manipulating the MAPK pathway in host cells; among such drugs is ergosterol peroxide, which has anti-PDCoV activity through this way [24,25]. The MAPK pathway includes three main signaling cascades. The extracellular signaling-regulated kinase (ERK) pathway is one of three. Activation of the ERK pathway also includes three signaling cascades (Raf, MEK1/2 and ERK1/2). ERK is a key component of cellular signal transduction and regulates cell functions and viral infections [25].

In this study, the effect of NAM on PEDV and PDCoV replication was identified. The results indicate that NAM negatively regulates PEDV and PDCoV infection in a dose-dependent manner, and its effects are independent of the virus titer and infection time. The antiviral effect of NAM is probably implemented by suppressing activation of the ERK1/2/MAPK pathway and blocking viral RNA synthesis. The results of this study are helpful for expanding potential application fields for NAM and for developing new therapeutic drugs for the clinical treatment of PEDV and PDCoV.

## 2. Materials and Methods

### 2.1. Cell Lines, Viruses and Drugs

Porcine kidney (LLC-PK1) cells (ATCC, CL-101), swine testis (ST) cells (ATCC, CRL-1746) and African green monkey kidney (Vero) cells (ATCC, CCL-81) were purchased from ATCC. LLC-PK1 and ST cells were cultured in Eagle’s minimal essential medium (MEM, Gibco, USA), and Vero cells were cultured in Dulbecco’s modified Eagle’s medium (DMEM, Gibco, USA). Both types of media were supplemented with 10% fetal bovine serum (Gibco, USA), and the cells were incubated at 37 °C under 5% CO_2_. PEDV strain CH/HBXT/2018 (GenBank No: MH816969) and PDCoV strain CH/XJYN/2016 (GenBank No: MN064712) were isolated and maintained in our laboratory. NAM was purchased from MACKLIN (China), dissolved in phosphate-buffered saline (PBS) and stored at −80 °C.

### 2.2. Cell Viability Assay (Cytotoxicity)

A cell viability assay of LLC-PK1, ST and Vero cells was performed in 96-well plates. Briefly, the cells were seeded into 96-well plates at 5000 cells per well. To detect the effect of NAM on cell viability, different concentrations of NAM were added to the 96 wells containing a cell monolayer, and eight replicates with each concentration of NAM were included in the plate. Cells treated with medium served as negative controls. After 48 h, the cells were washed twice with PBS, and 100 μL of medium with 3-(4,5-dimethylthiazol-2-yl)-2,5-diphenyltetrazolium bromide (MTT) (5 mg/mL) was then added to each well. After incubation for 4 h, the formazan crystals were carefully discarded. The reaction was stopped by adding 150 μL of DMSO, and the absorbance was measured by a plate reader at 590 nm. Cell viability (%) was determined as the percentage ratio of the absorbance of the NAM-treated samples versus that of the untreated control samples.

### 2.3. Quantitative Real-Time Reverse-Transcription PCR (RT-qPCR)

RNA was extracted from virus stock solution or cell lysates using TRIzol reagent (TaKaRa, Dalian, Japan) according to the manufacturer’s instructions. Absolute RT-qPCR targeting PEDV N and PDCoV N mRNA from virus stock solution was performed as described previously [26,27]. Relative RT-qPCR targeting *PEDV N*, *PDCoV N*, *C/EBPa* and *C/EBPβ* mRNA in the cell lysates was performed.

### 2.4. Western Blotting

Protein lysates were obtained from cells using ice-cold RIPA lysis buffer. The protein samples were subjected to SDS–PAGE and transferred to polyvinylidene fluoride (PVDF) membranes (Millipore, Boston, MA, USA). The membranes were blocked with 5% skimmed milk for 1 h at room temperature and then incubated with primary antibodies (anti-PEDV N MAb 1:5000; anti-PDCoV N MAb 1:5000; anti-β-actin MAb 1:3000; anti-β-tubulin MAb 1:3000; anti-ERK1/2 MAb 1:1000; anti-pERK1/2 MAb 1:2000) at room temperature for 2 h or 4 °C overnight. Subsequently, the membranes were subjected to four 10-min washes and incubated with HRP-conjugated secondary antibodies (goat anti-mouse IgG HRP 1:5000; goat anti-rabbit IgG HRP 1:5000) for 1 h at room temperature. Protein bands were detected using an enhanced chemiluminescence system (Thermo, Waltham, MA, USA).

### 2.5. Indirect Immunofluorescence Assay (IFA)

The cells were seeded into 12-well plates, incubated for 12 h and exposed to different concentrations of NAM for 1 h. Subsequently, the cells were infected with viruses and exposed to the corresponding concentration of NAM for 24 h. The cells were then fixed with 4% paraformaldehyde, permeabilized with 0.1% Triton X-100 and blocked with 3% bovine serum albumin (BSA). The cells were incubated with mouse anti-PEDV N MAb (1:2000) primary antibody for 2 h at room temperature. After three washes with PBS, FITC-conjugated goat anti-mouse (1:2500) was added, and the cells were incubated for 1 h at room temperature. After three more washes with PBS, the cells were stained with DAPI (1:2000) for 10 min, washed three times with PBS at room temperature and observed under a confocal microscope.

### 2.6. Investigation of the Effects of NAM on the Viral Replication Cycle

The effects of NAM on different stages of the PEDV and PDCoV replication cycles were compared. The cells were plated into 35 cm^2^ dishes, and the following four experiments were carried out: (1) Attachment: Cells were pretreated with or without NAM at the corresponding concentration (Vero cells for PEDV infection were treated with 0.5 mM; PK cells for PDCoV infection were treated with 0.25 mM) at 37 °C for 1 h, precooled at 4 °C for 0.5 h and then inoculated with the virus (PEDV or PDCoV, MOI = 50) at 4 °C for 1 h to avoid internalization of the virus. The cell medium was then discarded, and the cells were washed three times with PBS. The cell lysates were collected to determine the number of viral copies by real-time RT-qPCR. In the other group of experiments, the same protocol was first used, but ultimately the old cell medium containing NAM or not was discarded. New maintenance medium without NAM was added to the dishes after washing three times. Whole-virus samples were stored at −70 °C and used for the TCID_50_ assay. (2) Internalization: Cells cultured in 35 cm^2^ dishes were infected with viruses (PEDV or PDCoV, MOI = 50) at 4 °C for 1 h and washed three times to discard the nonadsorbed viruses, and the medium was replaced with corresponding medium with or without NAM. The cells were then incubated at 4 °C for 1 h to facilitate the effects of NAM and then at 37 °C for 1 h. The supernatant was discarded, and the cells were washed three times. Trypsin was used to digest the cells. The cells were collected, and the number of viral copies was measured by RT-qPCR. In another set of experiments, the digested cells were resuspended in maintenance medium, after which the viral titers were determined by the TCID_50_ method. (3) Replication: Cells were infected with virus (PEDV or PDCoV, MOI = 10) at 37 °C, and at 2 h postinfection (hpi), the cell supernatants were removed and cultured in fresh maintenance medium with or without NAM. The cells were cultured at 37 °C for 7 h and washed three times. The infected cell lysates or whole-virus samples were collected after new maintenance medium without NAM was used to replace the culture medium for RT-qPCR or TCID_50_ analyses. (4) For release, cells were infected with virus for 10 h, and the cell supernatants were removed and cultured in fresh medium with or without NAM for 2 h for NAM pretreatment. The cell supernatants were removed, and fresh medium with or without NAM was added for 1 h. The cell supernatants were harvested and titrated using the TCID_50_ method.

### 2.7. NAM Antiviral Activity Assay

The effects of NAM treatment at various stages of viral infection (MOI = 0.01) were compared in LLC-PK1 and Vero cells to investigate the inhibitory effect on PEDV or PDCoV infection at different stages. In brief, cells were seeded into 6-well plates, and the following three experiments were carried out: (1) Cells in the first group, which was named the pretreatment group, were exposed to NAM for 1 h before viral inoculation. Subsequently, the cells were washed twice with PBS, and fresh maintenance medium was added to the plates. The cells were then incubated for 24 h. (2) Cells in the second group, which was named the cotreatment group, were infected with virus for 1 h and treated with or without NAM. After two washes, maintenance medium supplemented with NAM at the corresponding concentration was added, and the cells were incubated for 24 h. (3) Cells in the last group, which was named the posttreatment group, were infected with virus at 37 °C for 1 h. The cells were washed twice, supplemented with maintenance medium with or without NAM and incubated. The cell lysates and cell culture supernatants were collected at 24 hpi. All groups were analyzed in three independent experiments. The antiviral activity of NAM was determined by RT-qPCR and TCID_50_ analyses as previously described.

### 2.8. Statistical Analyses

The results are expressed as the means ± standard deviations (SDs) from three independent experiments. Statistical analyses were performed using Student’s t test. Differences were considered significant if *p* < 0.05. Statistical significance is indicated in the figures as follows: * 0.01 < *p* < 0.05, ** *p* < 0.01.

## 3. Results

### 3.1. Minimal Cytotoxicity of NAM

**Figure 1:**
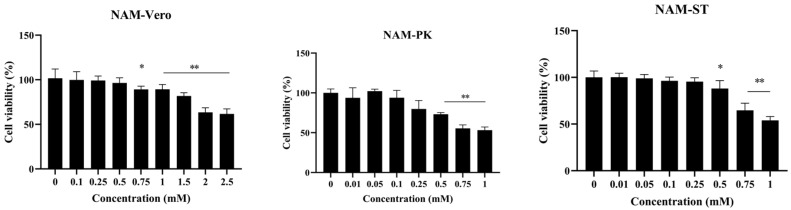
Evaluation of the cytotoxicity of NAM in Vero, PK and ST cells. Cell viability was meas uredat 48 h after treatment using an MTT assay. OD readings of duplicate samples were plotted. Three independent experiments were performed. The data are presented as the means ± standard deviations (SDs) of eight duplicate samples. Differences were considered significant if (*) 0.01 < *p* < 0.05 and (**) *p* < 0.01.

To investigate the potential cytotoxicity of NAM, cells treated with NAM for 48 h were tested by the MTT assay. The cytotoxicity of NAM in a concentration range of 0.1–2.5 mM or 0.01–1 mM was evaluated in three independent experiments. The results showed that the cytotoxic effects of NAM in Vero, PK and ST cells were dependent on dose. No obvious cytotoxicity was observed in Vero cells treated with NAM at concentrations below 0.5 mM, whereas NAM at concentrations up to 0.25 mM exhibited no cytotoxicity in LLC-PK1 and ST cells (Figure 1). Consequently, a concentration of 0.5 mM was selected for the subsequent studies with Vero cells unless otherwise indicated, and 0.25 mM was selected for the experiments with PK and ST cells.

### 3.2. NAM Exhibits Antiviral Activity against PEDV

**Figure 2:**
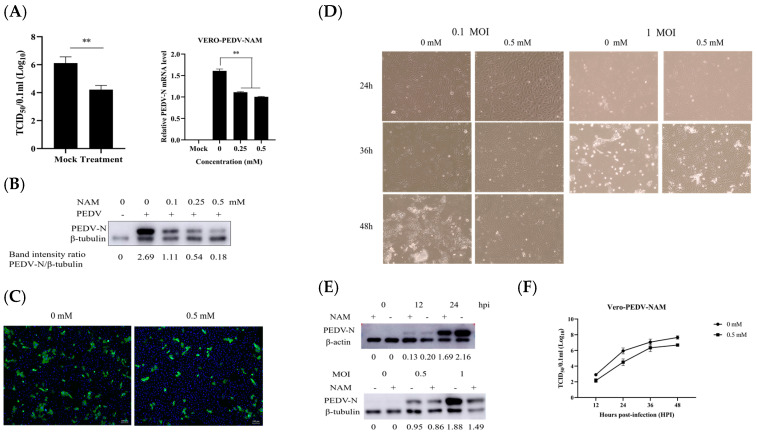
Antiviral activity of NAM against PEDV infection in Vero cells. (**A**) The inhibition of virus replication in Vero cells treated with 0.5 mM NAM for 24 h was evaluated by the TCID_50_ method. (**B**) The cells were incubated with NAM at different concentrations for 24 h. The effects were measured by Western blotting and RT-qPCR. (**C**) Virus replication in Vero cells treated with 0.5 mM NAM for 24 h was evaluated by IFA. (**D**,**E**) The inhibition of virus replication in Vero cells treated with NAM at different infection doses and times was assessed. (**F**) Growth curves with or without NAM treatment were detected by the TCID_50_ method. The data are presented as the means ± standard deviations (SDs) of eight duplicate samples. Differences were considered significant if (**) *p* < 0.01.

Vero cells were treated with 0.5 mM NAM throughout the PEDV infection cycle to evaluate whether NAM has a positive or negative effect on the production of PEDV. Cells were preincubated with NAM for 1 h and then infected with PEDV (MOI = 0.1) in the presence of 0.5 mM NAM. The viral yields after 24 h were assessed using different experimental methods. TCID_50_ assays performed using whole-virus lysates revealed that NAM decreased the production of PEDV from 5.47 to 4.43 (Figure 2A). Vero cells were then treated with different concentrations of NAM. The effect was evaluated by RT-qPCR and Western blotting (Figure 2B). Cell lysates were collected to detect the PEDV genomic mRNA and the expression of the viral nucleocapsid (N) protein. NAM significantly decreased the mRNA and protein expression of PEDV-N. The treatment of Vero cells with NAM led to a gradual, dose-dependent decrease in PEDV N protein levels. IFA was used to measure the expression of the PEDV N protein (Figure 2C). Viral propagation at 24 h after PEDV infection was then measured by IFA. In addition, the NAM treatment groups differed from the nontreatment group in terms of multiple and aggregated lesion effects. To further confirm the influence of NAM on PEDV replication, the effect of NAM treatment on replication was also studied using different infection times or MOIs (Figure 2D,E). We compared the cytopathic effect (CPE) of PEDV in normal medium and NAM-supplemented medium. Almost all cells infected with PEDV (MOI = 0.1) in the absence of NAM produced a CPE at 48 hpi, whereas cells in the NAM treatment group had just started to form lesions. An obvious difference was found between the groups. Vero cells were infected with PEDV at an MOI of 1. Almost all cells in the absence of NAM had shed at 36 hpi, and the proportion of lesions composed of cells in the NAM treatment group was approximately 20%. The inhibitory effect was not affected by the infection time or dose. The growth curve was measured and indicated that NAM treatment resulted in a significant reduction in PEDV production. In particular, the TCID_50_ per 100 microliters at 24 hpi decreased from 6.065 to 4.335.

### 3.3. NAM Exhibits Antiviral Activity against PDCoV Infection

**Figure 3:**
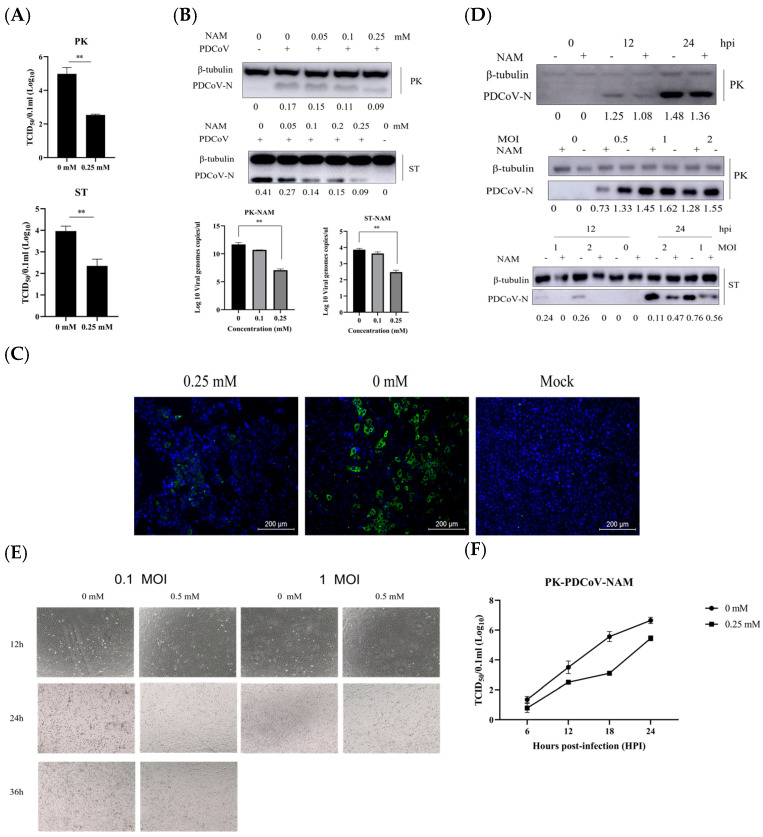
Antiviral activity of NAM against PDCoV infection in LLC-PK1 and ST cells. (**A**) The inhibition of virus replication in LLC-PK1 cells treated with NAM at 0.25 mM for 24 h was evaluated by TCID_50_ analysis. (**B**) The effect of the incubation of LLC-PK1 and ST cells with NAM at different concentrations on the replication of PDCoV at 18 hpi was assessed by Western blotting and RT-qPCR. (**C**) NAM was added, and the cells were incubated for 18 h. The effect was measured by IFA. (**D**) The effect of NAM treatment on the replication of PDCoV at different infection times and with different doses in LLC-PK1 and ST cells was assessed by Western blotting. (**E**) The inhibition of virus replication in Vero cells treated with NAM at different infection doses and times was assessed. (**F**) Growth curves with or without NAM treatment were detected by the TCID_50_ method. The data are presented as the means ± standard deviations (SDs) of eight duplicate samples. Differences were considered significant if (**) *p* < 0.01.

PDCoV is a member of the swine intestinal coronavirus family, and the effect of NAM treatment on the replication of PDCoV was also investigated. LLC-PK1 and ST cells were treated with 0.25 mM NAM throughout the PDCoV infection cycle, including a pretreatment step for 1 h before PDCoV infection, and the cells were then infected with PDCoV (MOI = 0.1). The viral yields after 24 h were assessed by different experimental methods. The whole-virus lysate was collected for the TCID_50_ assay. Accordingly, 0.25 mM NAM treatment resulted in a reduction in the viral yield. The results revealed that NAM decreased the production of PDCoV in both cell lines. The TCID_50_ value decreased from 4.98 to 2.54 in LLC-PK1 cells, and the ST ranged from 3.96 to 2.35 log10 (Figure 3A). The cells were treated with different concentrations of NAM. First, the cells were pretreated with the corresponding concentration of NAM for 1 h. The cells were infected with PDCoV in the presence of the corresponding NAM concentration (MOI = 0.1). The effect was evaluated by Western blotting and RT-qPCR. The cell lysates were collected at 18 hpi for the assessment of viral RNA and protein expression (Figure 3B). The effect of NAM on PDCoV infection was evaluated. Similar results to those obtained for PEDV were found for the PDCoV groups. NAM decreased the amounts of genomic PDCoV mRNA and PDCoV-N protein. NAM treatment led to a gradual, dose-dependent decrease in PDCoV N protein expression. The above-mentioned inhibitory effect was independent of cell type, and the LLC-PK1 and ST results were consistent. The RT-qPCR results showed that the group treated with NAM (0.25 mM) showed significantly inhibited viral replication compared with that of the untreated group. Accordingly, 0.1 mM and 0.25 mM NAM induced a 0.975 and 4.615 log reduction in LLC-PK1 and a 0.235 and 1.385 log reduction in ST, respectively. To determine the influence of time or MOI on PDCoV replication, we also assessed replication at different times or upon infection at different MOIs (Figure 3C). The results showed that NAM inhibited PDCoV replication at different times and MOIs. Viral propagation at 18 h after PDCoV infection was then measured by IFA (Figure 3D). The treatment of LLC-PK1 cells with 0.25 mM NAM led to a decrease in PDCoV N protein expression. To further confirm the influence of NAM on PDCoV replication, the CPE was observed in LLC-PK1-infected PDCoV treated with NAM at different times or MOIs (Figure 3E). The above results were the same as those in Vero cells infected with PEDV. The growth curve indicated that NAM inhibited the replication of PDCoV (Figure 3F).

### 3.4. NAM Inhibits the Synthesis of Viral RNA

**Figure 4:**
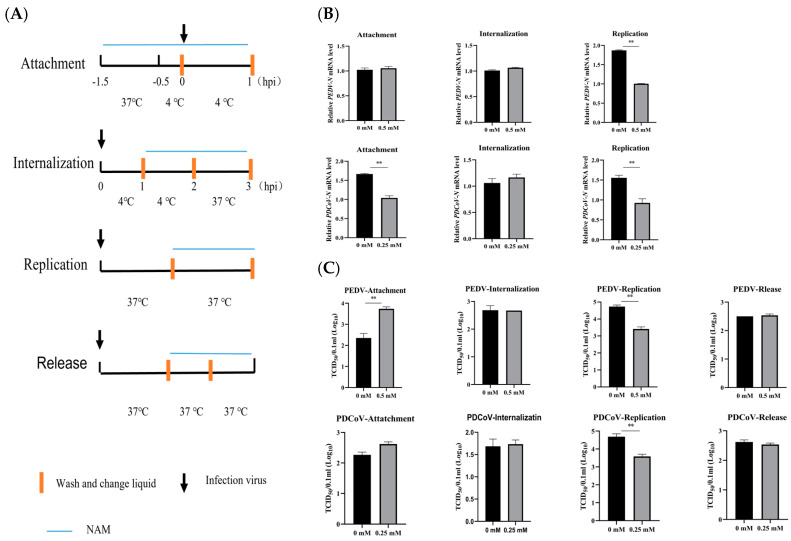
The antiviral effect of NAM on the viral replication cycle was measured by RT-qPCR and TCID_50_ analysis. (**A**) Virus-infected cells were treated with NAM at different stages of the life cycle. (**B**) RT-qPCR was used to assess the effect of NAM on the N mRNA level of PEDV-infected Vero cells and PDCoV-infected LLC-PK1 cells at different replication stages. (**C**) The effect of NAM on virus-infected cells at different replication stages was evaluated by the TCID_50_ method. The data are presented as the means ± standard deviations (SDs) of eight duplicate samples. Differences were considered significant if (**) *p* < 0.01.

To further study which steps of the viral life cycle are affected by NAM treatment, NAM was added to the cells during different stages of virus infection: attachment, internalization, replication and release (Figure 4A). According to the results described above, 0.5 mM and 0.25 mM NAM were selected for subsequent PEDV and PDCoV infection experiments, respectively. The results are shown in Figure 4B,C. The RT-qPCR and TCID_50_ assay results showed that both the RNA level and viral titer in the NAM treatment group were similar to those in the virus-only internalization and release groups. NAM treatment had no significant effect on the process, as revealed by the lack of a significant difference between the treated groups and the untreated group. In the attachment experiments, medium with or without NAM had an effect on the virus. RT-qPCR results showed that NAM significantly inhibited PDCoV attachment to LLC-PK1 cells. However, the TCID_50_ results were the opposite without statistical significance. NAM also increased the attachment of PEDV to Vero cells. However, the difference in performance between the two groups was nonsignificant. Moreover, NAM treatment had an obvious inhibitory effect on RNA synthesis. Both viruses showed consistent results. The mRNA levels of PEDV N and PDCoV N in the replication group were decreased to nearly one-half and two-thirds that in the nontreatment group, respectively. A TCID_50_ assay using whole-virus lysate revealed that NAM decreased the production of PEDV from 4.73 to 3.42 (Figure 2A) and that of PDCoV from 4.685 to 3.6. In brief, NAM mainly inhibited PEDV and PDCoV infection during the replication phase of the virus life cycle.

### 3.5. Evaluation of the Therapeutic Effect of NAM

**Figure 5:**
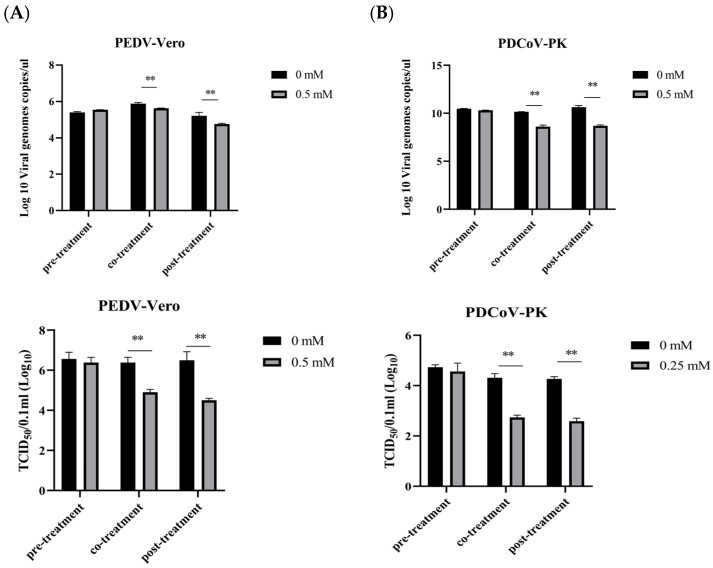
The therapeutic effects of NAM were evaluated by RT-qPCR and the TCID_50_ method, and the samples were taken from virus stock solutions. (**A**) Vero cells were infected with PEDV and treated with 0.5 mM NAM. (**B**) LLC-PK1 cells were infected with PDCoV and treated with 0.25 mM NAM. The data are presented as the means ± standard deviations (SDs) of eight duplicate samples. Differences were considered significant if (**) *p* < 0.01.

To evaluate the therapeutic effect of NAM during virus infection, NAM was added to medium before (pretreatment), during (cotreatment), and after (posttreatment) virus inoculation (Figure 5A). All analyses were performed 24 h after infection with virus at an MOI of 0.1, and 0.5 mM NAM was chosen for Vero cells. The virus was collected. In the pretreatment experiment, pretreatment with NAM resulted in only a slight, nonsignificant reduction in the number of viral genome copies during PEDV infection. NAM treatment inhibited virus replication in the cotreatment and posttreatment groups. NAM at the corresponding concentration clearly suppressed PEDV replication. In the cotreatment group, the viral genome copy per microliter was reduced by 0.245 log10, and the posttreatment group showed an approximately 0.46 log10 reduction. Upon infection, viral titers were measured. The viral titer per 100 microliters was determined to be reduced by 1.38 and 2.

The influence of NAM on PDCoV replication was also evaluated. All experiments were performed 24 h after infection. An MOI of 0.1, and 0.25 mM NAM were selected for the treatment of LLC-PK1 cells. Similar phenomena were also observed with NAM-treated PDCoV, and NAM pretreatment had no effect on PDCoV. NAM treatment inhibited virus replication in the cotreatment and posttreatment groups, which showed decreases in the viral genome copies per microliter of 1.545 and 1.925 log10, respectively. The viral titer per 100 microliters was determined to be reduced by 1.56 and 1.67, respectively.

### 3.6. Suppression of ERK1/2/MAPK Activity by NAM

**Figure 6:**
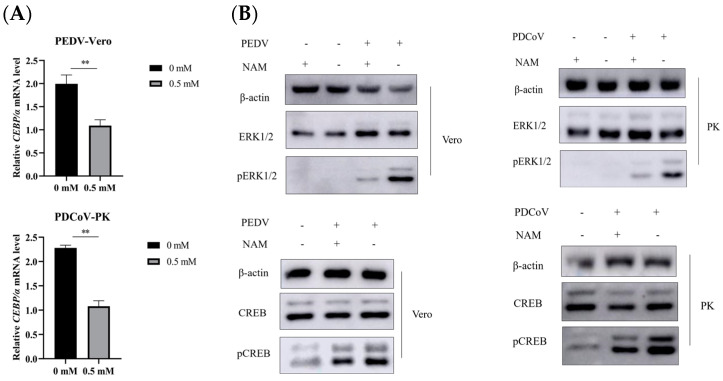
Effect of NAM on the ERK1/2/MAPK pathway in the context of viral infection. (**A**) The expression level of C/EBPa was measured by RT-qPCR. (**B**) Western blotting was used to detect the activation of ERK1/2 and CREB after incubation with NAM at 12 hpi with PEDV and PDCoV infection at an MOI of 0.1. The data are presented as the means ± standard deviations (SDs) of eight duplicate samples. Differences were considered significant if (**) *p* < 0.01.

We speculated that NAM may participate in regulating the transcription factors AP-1, C/EBPa and PPARa by affecting the activity of pERK1/2. To test this hypothesis, we evaluated the effect of NAM on the activity of pERK1/2 in Vero and LLC-PK1 cells in the context of viral infection. Therefore, C/EBPa was selected first for verification in Vero and LLC-PK1 cells to rule out the effect of cell type. RT-qPCR results showed that NAM treatment indeed inhibited the transcription of C/EBPa in Vero and LLC-PK1 cells (Figure 6A), and Western blot analysis showed that NAM treatment inhibited the activation of pERK1/2 (Figure 6B). These results showed that the transcription factors C/EBPa, PPARa, AP-1 and CREB were regulated by NAM. We chose another transcription factor named CREB to confirm that the signal was activated. As shown in Figure 6B, NAM inhibited the activity of pCREB. These data suggest that NAM may affect the expression of transcription factors through the ERK1/2 pathway. The replication of PEDV and PDCoV could be inhibited by NAM, and this effect may be partially dependent on the activation of the ERK1/2/MAPK pathway.

## 4. Discussion

Both PEDV and PDCoV are important pathogens that cause fatal diarrhea in piglets [5,6,7]. A global outbreak of PEDV had a significant impact on the international pig breeding industry in 2014, and this PEDV epidemic was later suppressed due to the emergence of vaccines. However, there is variation among PEDV strains, and the sequences of traditional strains substantially differ from those of variant strains [27,28,29,30]. Early vaccines showed limited protection against variant strains [28,29], and the PEDV epidemic returned [30]. In addition, PDCoV is a newly discovered zoonotic deltacoronavirus [4]. To date, relatively few studies have investigated PDCoV, and these studies have mainly focused on the isolation and culture of the pathogen. There are no commercial vaccines available for PDCoV, and there is no virus neutralization cross-reactivity between PEDV and PDCoV. Coronavirus is a single-stranded positive RNA virus that mutates easily. The sequences of different variant strains are complex and diverse, and their antigenicity may be different [31]. This requires the development of alternative strategies to preemptively prevent infection while developing vaccines. The development of antiviral drugs is considered an effective way to control corresponding diseases. For example, in the 2019 global outbreak of COVID-19, which is caused by infection with another coronavirus, the virus was initially treated with antiviral drugs because no vaccine was available. More recently, COVID-19 was found to be resistant to traditional mutation-induced vaccines that could not achieve complete protection, and antiviral drugs were therefore also utilized. Therefore, the research and development of antiviral drugs for mutable strains are crucial. The threat of new and re-emerging coronaviruses highlights the need to develop broad-spectrum antiviral drugs [32].

In this study, we found that NAM treatment inhibited the replication of PEDV and PDCoV in a dose-dependent manner, and this inhibitory process was not related to the virus dose or infection time. On the basis of life cycle, four phases, namely, adhesion, internalization, replication and release were investigated. NAM treatment had no obvious effect on the number of PEDV or PDCoV nucleoproteins at the early stages of replication, but had a substantial and apparent effect at the RNA synthesis stage. It is possible that viruses are strictly intracellular parasitic pathogens, and their replication depends entirely on host cells. Viral replication consumes a large quantity of host energy, which significantly alters host metabolism. As a key component of the tricarboxylic acid cycle, NAM may participate in the regulation of virus replication by regulating cell metabolism [33]. At the early stage of virus replication, namely, when the virus requires relatively little energy, cell metabolism has a marginal impact on adhesion and internalization. Similarly, virus release occurs in the same manner. Therefore, NAM treatment had no significant effect on the processes of virus attachment, internalization and release compared with the results obtained with the untreated group. However, in the RNA synthesis process, viruses need to consume a large amount of host energy, so the influence of NAM on this stage regulates virus replication. In addition, NAM has certain effects on oxidative stress, inflammatory damage and immune function [15]. NAM prevents PARP degradation and directly inhibits caspase 3 for DNA repair. PARPs and SIRT, two classes of NADases, play key roles in DNA repair [34]. Activation of PARPs can contribute to decreases in the virulence and replication of the virus [35,36]. PARP1 activity is dependent on NAD+ availability [37]. Therefore, supplementation with NR and NAM significantly reduced the replication of coronavirus in mice sensitive to PARP activity [38]. Some researchers have chosen NMN to supplement NAD+ for its anti-inflammatory and antiviral effects. NAM and NMN are the precursors of NAD^+^. Several studies have found that combined treatment with NAD+ and its intermediate (NMN) could protect 30% of SARS-CoV-2-infected mice from death. In vivo mouse studies support a prospective clinical trial in which patients with COVID-19 could be treated by targeting the NAD+ pathway [23]. In human cells, NMN is mainly synthesized by NAM and phosphoribosyl pyrophosphate (PRPP) through NAM in the NAD+ salvage pathway [39]. Viral replication can cause significant DNA damage in a cell. Therefore, PARP is activated to inhibit viral replication.

We determined that NAM can be used as a treatment for PEDV and PDCoV infection, but has no preventative effect. The role of NAM in the regulation of replication may mainly be achieved by affecting DNA damage, however, there is no DNA damage in the absence of viral infection, NAM does not work. Although the diseases caused by PEDV and PDCoV have similar clinical symptoms, these viruses belong to different coronavirus genera and encode different nucleotide sequences and amino acid sequences. NAM had an inhibitory effect on not only the alphacoronavirus PEDV and the deltacoronavirus PDCoV, but also the betacoronavirus SARS-CoV-2. Moreover, based on the results of this study, we speculate that NAM may have broad antiviral activity against coronaviruses, and the higher the replication rate of the virus is, the more obvious the inhibitory effect of NAM will be.

NAM has been shown to be involved in the regulation of viral replication by influencing host cell metabolism [23,35,36,37]. In addition, some studies have indicated that NAM is also related to some signaling pathways [40]. A study indicated that NAM can inhibit activation of the MAPK and AKT/NF-κB signaling pathways. MAPK cascade activation is at the center of multiple signaling pathways, such as insulin receptor signaling; the Warburg effect; hypoxia signaling; and the mTOR, AMPK and some other metabolism-related signaling pathways. As a common pathway involved in the viral infection of cells, various viruses have been found to regulate MAPK to increase their production [24,25]. ERK is a key component of the cellular signal transduction pathway that regulates a variety of cellular functions, and has been proven to regulate a variety of viral infections [24,25]. Studies have also shown that the ERK signaling pathway plays an important role in the life cycle of PEDV and PDCoV, and is thus beneficial to viral infection [25,41]. Moreover, it has been found that valinomycin can inhibit the replication of PEDV by changing activation of the MAPK pathway [42]. PERK is a key metabolic hub for the immunosuppressive function of microphages [43]. Inhibition of PERK can inhibit the immunosuppressive activity of macrophages. NAM may also inhibit cell metabolism and the inflammatory response during viral replication by inhibiting ERK activity. Previous studies conducted by other research groups have shown that NAM inhibits the expression of C/EBPa, PPARa and AP-1 in HepAD38 and HepG2.2.15 cells [20]. In addition to the ERK1/2 gene, C/EBPa and CREB were selected to confirm that NAM regulates ERK1/2 activity in Vero and PK cells. C/EBPa and CREB are the main downstream transcription factors in the ERK1/2 pathway. The MEK1/2-ERK1/2 signaling pathway is well known for its important role in regulating gene expression [25]. PRRSV infection induces SOCS1 and SOCS3 expression through the AP-1 signaling pathway, and promotes immune escape [44,45]. Hu showed that miR-141 reduces the activity of the HBV promoter by downregulating PPARA, and thereby inhibits HBV replication [46]. In conclusion, this study shows that NAM treatment has antiviral activity in the replication phase against PEDV and PDCoV, and that the replication of PEDV and PDCoV is inhibited by NAM in a manner partially dependent on the ERK1/2/MAPK pathway. However, further study is required to elucidate the detailed mechanisms.

Compared with most drugs, NAM is a relatively weak inhibitor, and this nutrient has been widely produced and is widely used due to its nontoxicity, low cost and oral administration route. NAM is present in food and can also be synthesized in the human body. NAM can also be used as a low-concentration vitamin, nutritional supplement or drug. As with any drug, the use of NAM needs to be monitored for related side effects. Several studies have found that the IC50 value of NAM is 21.5 mM, and noted that NAM treatment has dose-dependent effects. At doses near 5 mM, NAM treatment exerts cellular protective effects, improving the viability and replication potential of cells in culture, whereas at doses above 20 mM, NAM causes apoptotic death [47]. Other researchers have also proposed that NAM at micromolar doses appears to exert cell-protective effects, whereas NAM at millimolar doses induces cell death, which is likely related to oxidative stress [48]. Thus, in conclusion, NAM at an appropriate dose can be used as an ideal antiviral drug for production.

## 5. Conclusions

In summary, we found that NAM treatment significantly inhibited PEDV and PDCoV reproduction, mainly during the replication phase of the virus life cycle. Moreover, antiviral effects against virus replication were observed in the cotreatment and posttreatment groups. The activity of ERK1/2 in NAM-treated cells was significantly downregulated. These data suggest that NAM treatment inhibited the replication of PEDV and PDCoV, likely by downregulating the expression of transcription factors through activation of the ERK1/2/MAPK pathway. Overall, this study is the first to suggest that NAM might be not only an important antiviral factor for swine intestinal coronavirus, but also a potential candidate to be evaluated against other human and animal coronaviruses.

## Data Availability

Not applicable.
